# Immune modulation to treat Alzheimer’s disease

**DOI:** 10.1186/s13024-025-00828-x

**Published:** 2025-03-31

**Authors:** Michael R. Duggan, David G. Morgan, Brittani R. Price, Binita Rajbanshi, Alfonso Martin-Peña, Malú Gámez Tansey, Keenan A. Walker

**Affiliations:** 1 Laboratory of Behavioral Neuroscience, National Institute on Aging, Intramural Research Program, Baltimore, MD 21224 USA; 2 Department of Translational Neuroscience, College of Human Medicine, Michigan State University, Grand Rapids, MI 49503 USA; 3 GE Health Care, Marlborough, MA 01752 USA; 4 Department of Neurology, Weill Institute for Neurosciences, University of California San Francisco, San Francisco, CA 94158 USA; 5 Department of Neuroscience, College of Medicine, University of Florida, Gainesville, FL 32610 USA; 6 McKnight Brain Institute, University of Florida, Gainesville, FL 32610 USA

## Abstract

Immune mechanisms play a fundamental role in Alzheimer’s disease (AD) pathogenesis, suggesting that approaches which target immune cells and immunologically relevant molecules can offer therapeutic opportunities beyond the recently approved amyloid beta monoclonal therapies. In this review, we provide an overview of immunomodulatory therapeutics in development, including their preclinical evidence and clinical trial results. Along with detailing immune processes involved in AD pathogenesis and highlighting how these mechanisms can be therapeutically targeted to modify disease progression, we summarize knowledge gained from previous trials of immune-based interventions, and provide a series of recommendations for the development of future immunomodulatory therapeutics to treat AD.

## Introduction

Despite major advances, dementia remains a primary cause of disease and disability across the globe, where the 55 million currently affected older adults is expected to double over the next 20 years [1, 2]. Alzheimer’s disease (AD; which accounts for 60–70% of diagnoses [3]) is the leading cause of dementia, but our understanding of AD pathogenesis is incomplete [4]. The groundbreaking successes of anti-amyloid beta (Aß) monoclonal antibodies (mAbs) provide support for the amyloid-cascade hypothesis, yet their overall clinical impact remains limited: even with a near complete removal of Aß plaques, the effect of these treatments (although statistically significant) only slows disease progression by 20–30% [5, 6]. In addition, cerebral edema and microhemorrhages (referred to as amyloid related imaging abnormalities; ARIA) remain a prominent concern of these mAbs, especially for *APOE*4 carriers, who are at the greatest genetic risk for late-onset AD. Contributions of other pathological processes in AD have been well documented (e.g., large- and small-vessel cerebrovascular disease, Tar-DNA binding protein (TDP)−43 pathology etc. [7],), but a consensus has emerged from multiple lines of research that immune functions and neuroinflammation are core biological features of AD [8]. Over half of the AD risk genes are specific to the brain’s resident immune cells – microglia – or play a prominent role in immune signaling [9–11]; comprehensive post-mortem tissue collections show consistent microglial activation and reactive astrocytes in early- and late-stage AD pathology; and functional investigations in preclinical models have demonstrated the causal precedence of immune mechanisms in recapitulating clinical AD characteristics [12–16].

Given the central role of immune function in AD, along with the modest therapeutic efficacy and potential side effects of recently approved mAbs, immunomodulatory therapeutics (defined here as therapeutic approaches that directly target immune cells or immunologically relevant molecules) represent a class of drugs with the unique potential to further slow the progression of AD. This review provides an overview of immunomodulatory therapeutics currently being developed for AD, including small molecules, biologics, novel compounds, and re-purposed drugs. Based on a comprehensive review of the literature and publicly available clinical trial information, this review prioritizes discussion of immune-based therapeutics that are in the later stages of development and/or have more peer-reviewed results (Table 1). Additionally, this review summarizes the knowledge gained from unsuccessful trials of immune-based interventions, and provides a series of recommendations for the development of future immunomodulatory therapeutics in AD.

**Table 1:** Immunomodulatory therapeutics

Name	Type	Manufacturer	Stage	Repurposed?	MOA
AL002	Biologic	Alector	Phase 2	No	TREM2
VG-3927	Small molecule	Vigil Neurosciences	Phase 1	No	TREM2
VHB937	Biologic	Novartis	Preclinical	No	TREM2
CpG1018	Biologic	Dynavax Technologies	Phase 1	Yes	TLR9
Valacyclovir	Small molecule	GlaxoSmithKline	Phase 2 Phase 2	Yes	Antiviral
Emtricitabine	Small molecule	Gilead Sciences	Phase 1	Yes	Antiretroviral
Lamivudine	Small molecule	Strides Pharma	Phase 2	Yes	Antiretroviral
Censavudine	Small molecule	Transposon Therapeutics	Preclinical	Yes	Antiretroviral
AL044	Biologic	Alector	Phase 1	No	MS4A
Montelukast	Small molecule	IntelGenx	Phase 2	Yes	LTD4-CysLT
Edicotinib	Small molecule	Johnson & Johnson	Phase 1	No	CSF1R
XPro1595	Biologic	INmune Bio	Phase 2	No	sTNF
H-151	Small molecule	Tocris, MedChem, InvivoGen etc.,	Preclinical	Yes	cGAS-STING
ACI-6635	Biologic	AC Immune	Preclinical	No	NLRP3-ASCs
ATLX-1008	Biologic	Alchemab Therapeutics	Preclinical	No	CD33
Protollin	Biologic	I-MAB Biopharma	Phase 1	Yes	SCRA
IBC-Ab002	Biologic	ImmunoBrain Checkpoint	Phase 1	No	PD-1
Levetiracetam	Small molecule	UCB Pharma	Phase 2	Yes	SV2A
HT-ALZ	Small molecule	Hoth Therapeutics	Preclinical	Yes	NK-1
Lenalidomide	Small molecule	Celgene	Phase 2	Yes	CRBN
MW150	Small molecule	Neurokine Therapeutics	Phase 2	No	p38
Sargramostim	Biologic	Partner Therapeutics	Phase 2	Yes	JAK-STAT
Baricitinib	Small molecule	Eli Lilly	Phase 1	Yes	JAK-STAT
TB006	Biologic	TrueBinding	Phase 2	No	galectin
Aß-Gas6	Biologic	KAIST	Preclinical	No	Gas6
BMS-984923	Small molecule	Allyx Therapeutics	Phase 1	Yes	C1q-mGluR5
ANX005	Biologic	Annexon	Preclinical	No	C1q
COYA 301	Biologic	Coya Therapeutics	Phase 2	Yes	IL-2-Treg
Foralumab	Biologic	Tiziana Life Sciences	Phase 2	Yes	CD3-Treg

*Key*: *C1q* Complement component 1q, *CD3* Cluster of differentiation factor 3, *CD33 *Cluster of differentiation factor 33, *cGAS-STING* Cyclic GMP-AMP synthase-stimulator of interferon genes, *CRBN* Cereblon, *CSF1R* Colony stimulating factor 1 receptor, *Gas6* Growth arrest-specific 6, *IL-2* Interleukin-2, *JAK-STAT* Janus kinase/signal transducers and activators of transcription, *KAIST* Korean Advanced Institute of Science and Technology, *LTD4-CysLT* Leukotriene D4-Cysteinyl leukotriene, *mGluR5* Metabotropic glutamate receptor 5, *MS4A* Membrane-spanning 4A, *MOA* mechanism of action, *NK-1* neurokinin 1, *NLRP3-ASCs* NLR Family Pyrin Domain Containing 3- apoptosis speck-like complexes, *p38* p38 mitogen-activated protein kinases, *PD-1* Programmed cell death protein 1, *SCRA* Scavenger Receptor Class A, *SV2A* synaptic vesicle glycoprotein 2A, *sTNF* soluble Tumor Necrosis Factor, *TLR9* Toll-like receptor 9, *Treg* regulatory T-cell, *TREM2* Triggering receptor expressed on myeloid cells 2

## Immune mechanisms in Alzheimer’s disease

### Central nervous system immunity

There are two major branches of the immune system [17]. Innate immunity recognizes pathogens or tissue damage using pattern recognition receptors to detect general molecular features that are not found in healthy cells and tissues (i.e., pathogen associated molecular patterns, PAMPs; damage associated molecular patterns, DAMPs). Adaptive immunity is a flexible system that can be modified to recognize distinct molecules on specific pathogens, inhibit tissue damage related to particular infectious agents, and prevent re-infections (i.e., due to the development of immune memory). In general, innate immunity responds more quickly but with restricted specificity, while adaptive immunity responds more slowly but with greater specificity. Innate immune cells include monocyte, macrophages, dendritic cells, neutrophils, mast cells, basophils, eosinophils and natural killer cells. The adaptive immune system consists of a variety of T- and B-cells. While some immune cells are tissue-specific and reside in certain organs, others in circulation can readily cross blood vessels to infiltrate organ systems when prompted by tissue damage or infectious triggers.

The central nervous system (CNS) is immune-specialized due the blood–brain-barrier (BBB), where endothelial cells are joined by tight junctions (i.e., non-fenestrated capillaries) [18]. The brain’s primary resident immune cells, microglia, share functional similarities with monocyte-derived cells, but have a unique ontogeny as they originate from yolk sac progenitors. Border associated macrophages (which also have a unique lineage in that they derive from the yolk sac rather than the bone marrow) are located at the edges of the parenchyma, along blood vessels, the meninges, and the choroid plexus [19]. Other cells in the brain can also respond to immune signals and contribute to factors (e.g. cytokines) that regulate immune activity. For example, astrocytes modulate phagocytosis, BBB permeability, and glutamate uptake, all of which can contribute to neuroinflammation [20]. There is continued surveillance of the brain by peripherally circulating immune cells, but they require additional steps (referred to as diapedesis) to cross the endothelium and enter the CNS. Fenestrated capillaries in the dura mater, choroid plexus, and other circumventricular organs provide opportunities for direct interactions between the brain and peripheral immune systems [21]. The dynamic interactions of central and peripheral immune mechanisms have been detailed previously [22]. Notably, some immune cells located in CNS border regions can also be mixed with, and replaced by, myeloid-derived macrophages from the skull’s bone marrow [23].

### Innate and adaptive immunity in AD

Perhaps the most convincing support for the immune system’s mechanistic role in AD comes from genome-wide association studies (GWAS) [24, 25] that have causally implicated a large number of microglia or immune-related genes [26]. For example, Triggering receptor expressed on myeloid cells 2 (TREM2) was discovered as an AD risk gene in 2013. A lack of *TREM2* activation is thought to hinder the microglial response to Aβ deposition. *TREM2* loss of function variants are second only to *APOE* in the strength of associations with AD risk [27–29].

Along with broader patterns of neuroinflammation, studies have identified specific innate immune mechanisms that influence AD pathogenesis. Single-cell transcriptomics has revealed a unique microglia phenotype involved in neurodegenerative disease called disease associated microglia (DAMs) [30–32]. In the context of AD, microglia transition from a homeostatic state, largely surveilling the brain parenchyma, to a DAM phenotype that attempts to clear or compartmentalize Aß in a TREM2-dependent manner. NLRP3 (NOD-, LRR- and pyrin domain-containing protein 3) inflammasome activation in microglia is yet another innate immune pathway that can contribute to AD pathology, as increased levels of inflammasome components (e.g., caspase-1, interleukin-1-beta, apoptosis speck-like complexes; ASCs etc.,) have been observed in AD brain tissue, especially in microglia and astrocytes surrounding amyloid plaques [33, 34]. Colony stimulating factor 1 receptor (CSF1R) upregulation and increased microglia proliferation are also characteristics of AD brains [35, 36]. Aging and AD pathology have also been associated with increased levels of brain tissue retrotransposons, which can trigger prolonged innate immune system responses and contribute to neurodegeneration [37–39]. This occurs as a result of compromised repression of retrotransposable elements: pieces of viral-derived DNA that have incorporated themselves into the human genome, and with the help of cytosolic reverse transcriptase enzymes, can replicate their DNA via an RNA intermediate, a process that is normally repressed [40]. Additionally, astrocyte-specific processes can play a significant role in AD-related neuroinflammation, namely by producing inflammatory mediators (e.g., chemokines and cytokines), releasing toxic byproducts (e.g., reactive oxygen species and peroxidized lipids), and contributing to key neuropathogenic pathways (e.g., *APOE* and complement signaling) [41, 42].

Recent studies have also illuminated the role of specific adaptive immune processes in AD. Brains of AD patients can show increased numbers of T-cells, and cerebral spinal fluid (CSF) analyses have indicated these are derived from a distinct set of expanded T-cell receptor clones, suggesting some degree of antigen-based recruitment, infiltration, and/or activation [43, 44]. In 5xFAD mice bred without B- and T-cells, increased microglial activation, inflammatory cytokine expression, and increased Aß deposition are observed, each of which can be rescued by bone marrow transplantation or administration of intravenous immunoglobulin[45]. Supporting these findings, immune checkpoint inhibition with a Programmed cell death protein 1 (PD-1) antibody leads to both monocyte and regulatory T-cell infiltration into the brains of tauopathy mice, rescuing cognitive dysfunction [46, 47]. On the other hand, some preclinical evidence suggests PD-1 modulators may not affect Aß deposition, and an examination in a different tauopathy model found that as mice age, their T-cells clonally expand and accumulate near regions of high tauopathy. Furthermore, T-cell depletion with anti-T-cell antibodies reduces neurodegeneration, as does depletion of microglia, which in turn prevents T-cell infiltration, suggesting that microglial dependent recruitment of T-cells into the brain may drive neurodegeneration [48, 49]. Conversely, PD-1 expression has also been shown to direct brain-resident CD8 + T cells toward a regulatory phenotype that restricts AD pathology and promotes microglial phagocytosis of Aß [50, 51]. These findings highlight how similar immune mechanisms may be both protective and deleterious in AD and indicate the need for targeted (rather than generalized) approaches to regulating neuroinflammation.

Despite growing knowledge of the immune system’s mechanistic contributions to AD, the role of neuroinflammation in AD progression is complex and not fully understood. While key components of AD, such as Aß and tau NFT’s, have been well characterized across the disease course, the field still lacks a comprehensive understanding of how the neuro-immune response to AD evolves across this time. It has become clear that immune function can contribute to AD pathogenesis in early preclinical, prodromal, and later stages of the disease process [8]. However, the relative contribution of central and peripheral immunity to neuroprotection and neurodegenerative processes across this multi-decade disease course remains unclear, as does the extent to which this immunologic contribution is modified by host factors, such as sex, comorbidities, and genetic background [18]. This understanding is complicated by the fact that distinct immune mechanisms likely influence different stages of AD pathogenesis, and similar immune mechanisms may have contrasting effects across different AD stages. Thus, similar to recently approved Aß-mAbs whose treatment efficacy can be dependent on disease stage, immune-based approaches will need to consider treatment timing and disease course in order to maximize therapeutic potential [5]. Although preclinical studies of immunomodulatory therapeutics have already yielded promising results, a more complete understanding of the complexities of CNS immunity will be essential for guiding the development of effective AD immunomodulatory strategies.

### Insights from existing therapeutics

Although recently approved mAbs (i.e., donanemab, lecanemab) may be considered primarily amyloid-targeting therapies, their inherent mechanisms and ARIA side effects further highlight the role of immune processes underlying AD. By binding to Fc receptors, they induce microglia-mediated phagocytosis and endosomal/lysosomal degradation of Aß [52, 53]. *APOE4*, a key regulator of neuroinflammation, is not only a leading risk factor for mAb-induced ARIA, but ARIA itself may be a byproduct of aberrant immune activation*.* Reminiscent of cerebral amyloid angiopathy (CAA)-related inflammation, ARIA may be caused a build-up of vascular amyloid that recruits immune cells (especially perivascular macrophages) in a complement-dependent manner, which then drives severe inflammation in the context of Aß-targeting mAbs, compromises BBB integrity, and induces ARIA [54–56].

## Lessons from immunomodulatory therapeutics that failed to meet their endpoints

Current immunomodulatory therapeutics build upon lessons learned from earlier interventions, incorporating these insights to refine and improve therapeutic strategies. Attempts to treat AD with broad anti-inflammatory or immunosuppressive drugs represent a response to dozens of observational and pharmacoepidemiologic studies that demonstrated an association between long-term use of anti-inflammatory medication and a lower AD risk [57, 58, 59]. In particular, non-steroidal anti-inflammatory drugs (NSAIDs) have been consistently associated with reduced AD risk in prospective studies, with few exceptions [59, 60]. Tumor necrosis factor (TNF) inhibitors have also been associated with reduced AD risk in large epidemiological studies, including a recent retrospective analysis that examined health records of 56 million individuals diagnosed with inflammatory diseases (e.g., rheumatoid arthritis) who were treated with TNF blockers [61, 62].

Multiple randomized controlled clinical trials (RCT) for NSAIDs (e.g., ibuprofen [63], naproxen [64]), which typically last 6 to 24-months and target patients with early AD dementia, have been uniformly negative. One prevention trial in mild cognitively impaired (MCI) patients (*n* = 1,457) found that the NSAID rofecoxib accelerated progression to AD [65], suggesting that dampening inflammatory responses during the AD prodrome may accelerate the disease. The Alzheimer’s Disease Anti-inflammatory Prevention Trial examining the efficacy of two NSAIDs (naproxen and celecoxib) in cognitively unimpaired older adults (*n* = 2,625) was prematurely halted due to concerns of excessive cardiovascular risk in participants treated with celecoxib. Examination of the two-year follow-up data indicated that participants taking celecoxib and naproxen had greater risk for progression to AD compared to placebo [66]. However, later examination of this study’s five-year follow-up data found a trend toward reduced AD risk and less CSF-defined neurodegeneration among participants in the naproxen arm [67]. Although the authors cautiously suggested that naproxen may reduce AD risk in individuals who begin taking the drug before the onset of cognitive impairment, a more recent trial which examined the effect of a two-year naproxen treatment in cognitively unimpaired participants with a family history of AD (*n* = 195) found that the naproxen-treated group did not differ from placebo on a multimodal composite score of cognitive, neuroimaging, and CSF measures [68]. Thus, clinical trials suggest that a broad reduction of inflammation or general immunosuppression using steroids or NSAIDs is not a viable strategy for reducing AD risk or slowing cognitive decline. One possible explanation is that the brief duration and typically later-life NSAID treatment of prior clinical trials may not recapitulate the therapeutic benefits associated with extended NSAID exposure beginning well before the onset of MCI or dementia.

Compared to this previous generation of immune-based therapeutics for AD, the set of therapies described below are distinct in three ways. First, as opposed to broad immunosuppression, these strategies typically focus on a single molecular pathway to boost protective (and/or dampen pathogenic) immune processes. Second, a greater proportion of the recent immune therapies target brain-specific (typically microglial) immune mechanisms rather than peripheral (non-CNS) immunity. Third, recent approaches have expanded beyond traditional small molecules to incorporate alternative strategies, including antibody-based biologics.

## Immunomodulatory therapeutics in the pipeline

### TREM2 (AL002, VHB937, VG-3927)

TREM2, a myeloid cell receptor strongly implicated in AD GWAS, is the target of multiple AD pharmacotherapies, including activating monoclonal antibodies AL002 (Alector) and VHB937 (Novartis), as well as small molecule agonists such as VG-3927 (Vigil Neurosciences). Since its discovery, a great deal of effort has gone into understanding TREM2 biology, particularly its role in shaping the microglial response in AD. TREM2 activation can induce a protective microglia phenotype (e.g., DAM) that enhances phagocytosis, increases cell survival, and reduces amyloid burden [69]. However, TREM2’s role in AD risk is not straight forward. For example, TREM2 has also been shown to contribute to microglial senescence and the accompanying inflammation and cognitive decline in 5xFAD mouse model of amyloidosis [70]. Whereas most research supports TREM2’s role in microglial-base amyloid clearance, how microglial TREM2 may affect tau pathology is less clear. For example, multiple studies suggest that TREM2 exacerbates tau pathology and Aß-associated tau seeding [71, 72], yet other work indicates that TREM2 deficiency heightens tau pathology [73]. Such converse effects on amyloid and tau may limit the benefits of TREM2 therapeutics, especially given the potential for diffuse microglial activation throughout the brain rather than Aß-localized activation. Along with its full-length, cell-surface isoform, the exact role of soluble TREM2 (sTREM2) requires further elucidation. Generated through the cleavage of the TREM2’s ectodomain by metalloproteases and/or alternative TREM2 splicing, sTREM2 has been shown to reduce amyloidosis, tau hyperphosphorylation, and cognitive deficits in rodent models [74, 75]. This soluble form could also act as a decoy receptor for the unidentified ligand on amyloid plaques that is responsible for attracting TREM2, thus blocking signaling in microglia [76]. sTREM2 levels have also been associated with both faster and slower rates of amyloid progression in autosomal-dominant AD depending on disease stage, suggesting the biological interpretation of sTREM2 fluctuations and broader complexities of TREM2 biology require additional investigation to understand their potential mechanistic significance in AD [77].

**Fig. 1:**
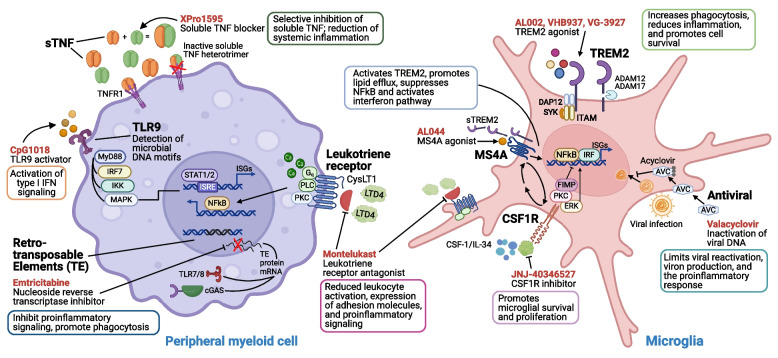
Proposed mechanisms of action for immunomodulatory therapeutics in Alzheimer’s disease (AD) clinical trials. Clinical stage immunomodulatory therapeutics currently being examined for AD target receptors on immune cells (e.g., peripheral myeloid cells, microglia) and immune mediators in circulation (e.g., sTNF). Drug names are listed in red. Below each drug name is the proposed mechanism of action. The immune pathways and processes affected by each drug are described in the adjacent box. *Abbreviations*: ACV, acyclovir; ADAM12, ADAM metallopeptidase domain 12; ADAM17, ADAM metallopeptidase domain 17; CSF1R, colony stimulating factor 1 receptor; DAP12, transmembrane immune signaling adaptor TYROBP; ERK, extracellular signal-regulated kinase; FIMP, chromosome 16 open reading frame 92; IKK, inhibitor of NF-kB kinase; IL-34, interleukin 34; IRF7, interferon regulatory factor 7; ITRAM, immunoreceptor tyrosine-based activation motif; MAPK, mitogen-activated protein kinase; MS4A, membrane spanning 4-domains; MyD88, myeloid differentiation primary response 88; NF-kB, nuclear factor kappa-light-chain enhancer of activated B cells; PKC, protein kinase C; sTNF, soluble tumor necrosis factor; STAT1, signal transducer and activator of transcription 1; STAT2, Signal transducer and activator of transcription 2; sTREM2, soluble triggering receptor expressed on myeloid cells 2; SYK, spleen associated tyrosine kinase; TLR9, Toll-like receptor 9; TNFR1, tumor necrosis factor receptor 1; TREM2, triggering receptor expressed on myeloid cells 2

TREM2 therapies are thought to stimulate the protective properties of microglia (e.g., phagocytosis of Aß; Fig. 1). A multi-center Phase I trial (NCT03635047) of AL002 was completed in 2020, where participants (*n* = 56) received a single intravenous injection of one of nine ascending doses or placebo, followed by lumbar puncture two days later and monitoring up to 12 weeks post-injection [78]. In addition to no serious adverse events, results showed AL002 induced a dose-dependent decrease in sTREM2 and a concomitant increase in soluble CSF1R. VHB937, another TREM2 antibody, boosts chemotaxis and phagocytosis in vitro, while results from AD, Parkinson’s disease (PD), and multiple sclerosis mouse models show it can protect neuronal viability, reduce neuroinflammation, and mitigate astrogliosis [79, 80]. In contrast to TREM2 activating antibodies, VG-3927 is a small molecule TREM2 agonist whose administration reduced plaque load and insoluble Aß_42_ levels in 5xFAD mice, while also inducing a DAM-like expression profile in microglia [81]. Another Phase 1 trial (NCT05450549) of a different TREM2 antibody, DNL919 (Denali Therapeutics), was initiated in 2021; however, its development was halted after treatment resulted in moderate anemia among several participants.

A Phase 2 trial of AL002 (NCT04592874) was initiated in 2021 in participants with early AD (*n* = 328), and recruitment was completed in 2023. Monthly injections (i.e., 15–60 mg/kg) will be administered over the course of 48, 72 or 96 weeks, with the Clinical Dementia Rating scale (CDR) as the primary endpoint. Secondary endpoints include cognitive measures, and exploratory endpoints include imaging and fluid biomarkers. Early in the trial, 3 participants presented with serious neurological adverse events in the form of ARIA, all of whom were *APOE4* homozygotes. After the protocol was amended to exclude *APOE4* homozygotes, no further serious adverse events have been reported [82]. During the revision of this article, AL002’s Phase 2 results were released, showing that it failed to meet its primary endpoint. AL002’s long term extension study has been halted, and its future status is unknown [83]. A Phase 1 trial (NCT06343636) of the small molecule VG-3927 is also currently underway and has an expected completion date of December 2024; single and multiple ascending oral doses will be administered orally to healthy participants (*n* = 90) with a focus on detecting adverse events. Preliminary data indicate VG-3927 can decrease sTREM2 levels [84].

### TLR9 (CpG1018)

Emerging strategies are examining the therapeutic potential of targeting TLR9. One such strategy uses CpG1018 (Dynavax Technologies), a short (i.e., 22-mer), unmethylated oligonucleotide sequence containing CpG motifs that have been used as an immunostimulatory adjuvant in several approved vaccines (e.g., hepatitis B virus; HBV) because it emulates bacterial DNA. Specifically, CpG1018 binds to TLR9 (Toll-like receptor 9; Fig. 1) during the co-presentation of a vaccine’s antigen, and boosts rates of antibody production [85–87]. CpG1018-TLR9 binding induces chemotaxis and subsequent Aß phagocytosis by murine microglia in vitro [88]. In 3xTg and Tg2576 AD mouse models, TLR9 stimulation with CpG1018 reduces amyloid pathology in brain regions associated with neurodegeneration (e.g., hippocampus) and preserves memory performance [89, 90]. Its beneficial effects have also been reported in murine and non-human primate models of vascular CAA [91, 92]. As CpG1018 does not cross the BBB, its exact mechanisms of action in AD remain unclear. Given that its capacity to enhance microglia mediated Aß clearance coincides with attenuated neuroinflammation despite elevated inflammatory cytokines in plasma, one possibility is that CpG1018-TLR9 binding on peripheral immune cells induces secretion of inflammatory mediators that can subsequently i) enter the CNS and directly activate microglial Aß clearance mechanisms, and/or ii) induce transient recruitment of peripheral immune cells to the CNS which then exert similar protective mechanisms (Fig. 1) [92]. Currently, subcutaneous CpG1018 injections are being evaluated in an 8-week, Phase 1 trial in Aß + PET participants (*n* = 39; NCT05606341), with an anticipated completion date of November 2024. Primary outcomes include adverse events, autoimmunity markers in blood, and ARIA; secondary outcomes include change in a battery of cognitive tasks as well as CSF and plasma Aß and tau measures.

### Antivirals (Valacyclovir)

Although the role of herpes viruses in AD is debated [93, 94], multiple large cohort studies suggest that herpetic infections, and viral infections more generally, can increase risk for AD and antiviral medications can mitigate such risk [95–97]. Accordingly, the therapeutic potential of valacyclovir, an antiviral treatment, is being examined for AD (Fig. 1). Valacyclovir (brand name Valtrex; GlaxoSmithKline; generically available since 2009) is a Food and Drug Administration (FDA)-approved, small molecule that is preferentially processed by thymidine kinases encoded by herpetic viruses. Given that valacyclovir also functions as an inhibitor of viral DNA polymerases, it has emerged as a widely used treatment for conditions like genital warts, cold sores, shingles, and chickenpox [98]. In a Phase 2 study (*n* = 32; NCT02997982), 4 weeks of oral valacyclovir (0.5 g/tri-daily/two weeks, 1.5 g/tri-daily/two weeks) increased intrathecal sTREM2 and Mini-Mental State Examination (MMSE) performance among HSV-infected *APOE4* carriers, although no change in CSF total tau or NfL were observed [99]. Two Phase 2 trials are currently assessing the efficacy of valacyclovir. In clinically defined AD and biomarker positive MCI cases who are HSV seropositive (*n* = 130; NCT03282916), an 18-month valacyclovir (4 g/daily) treatment will be examined with cognition as the primary outcome, PET-defined amyloid and tau as secondary outcomes, and cortical thinning, olfactory deficits, and antiviral titers as exploratory outcomes. In another trial among HSV seropositive individuals who either meet criteria for MCI or are AD biomarker positive (*n* = 50; NCT04710030), the effect of a 12-month valacyclovir treatment will be examined with respect to primary outcomes of amyloid PET, cognitive decline, and functional impairment. These studies, both lead by a group at New York State Psychiatric Institute, have anticipated completion dates of December 2024 and March 2025, respectively.

### Antiretrovirals (lamivudine, emtricitabine, censavudine)

Similar to the repurposing of antiviral agents, several approaches are also investigating the utility of antiretroviral compounds for treating AD. Lamivudine (brand name Epivir; ViiV Healthcare; generically available since 2014), emtricitabine (brand name Emtriva; Gilead Sciences), and censavudine (Transposon Therapeutics) are small molecule nucleoside reverse transcriptase inhibitors (NRTIs). Lamivudine and emtricitabine are widely used, FDA-approved treatments for human immunodeficiency virus (HIV) and HBV, two conditions in which reverse transcription plays an essential role. Both drugs inhibit viral replication and mitigate symptomology. Evidence from mouse tauopathy models and AD neural spheroids has shown that lamivudine can effectively suppress retrotransposon activation, attenuate neuroinflammation (especially TLR and cGAS activation), and preserve neuronal viability (Fig. 1) [100–102]. Such potent anti-inflammatory capacities have also been reported for other NRTIs, including emtricitabine, stavudine, and Kamuvudine-9, suggesting NRTIs may be considered as multi-modal therapies that are capable of mitigating AD pathology and restraining aberrant neuroinflammation [103–106]. In a recently completed Phase 2 trial of lamivudine among MCI participants (*n* = 12; NCT04552795), six months of oral treatment (0.3 g/daily) did not significantly alter cognition, but its effective penetrance of the CSF was linked to attenuated intrathecal markers of neuroinflammation (GFAP, FLT1) and increased plasma Aß_42/40_ [107]. A Phase 1 trial of emtricitabine in Aß + MCI and AD participants (*n* = 35; NCT04500847) was initiated in 2021 by Butler Hospital and Brown University, with an anticipated completion date of March 2024. In assessing 6 months of treatment (0.2 g/daily), the primary outcome will be adverse events, with secondary outcomes including inflammatory markers in blood, AD biomarkers in CSF, and cognitive functioning. Although no trials are currently registered, Transposon Therapeutics has also announced that it will expand the development of censavudine to investigate this drug in AD.

### MS4A (AL044)

Along with other biologic-based therapeutics for AD, AL044 (Alector) is an antibody that targets the MS4A (Membrane-spanning 4A) family of proteins, a group of transmembrane proteins that are expressed on microglia and associated with AD risk in GWAS [108, 109]. AL044’s mechanism of action is likely the stimulation of TREM2 and modulation of microglial homeostasis (Fig. 1). Along with in vitro data showing MS4A-targeting antibodies can induce corresponding alterations in sTREM2, MS4A variants associated with reduced AD risk and delayed dementia onset have been linked to higher TREM2 in plasma and higher sTREM2 in CSF [110, 111]. More recent data have showed these same protective variants also augment microglia interferon signaling, lipid metabolism, and cholesterol efflux [35]. Thus, boosting MS4A mediated signaling may enhance microglia capacities to respond to neuropathology and maintain a protective, anti-inflammatory phenotype. Although results of AL044 have not been peer reviewed, findings made available to investors suggest its intravenous administration can phenocopy the effects of protective MS4A gene variants, resulting in increased sTREM2 and membrane bound TREM2 in macrophages [112]. A 2022 press release announced the initiation of a Phase 1 trial for AL044, which sought to enroll healthy adults (*n* = 72) to assess the drug’s safety, pharmacokinetics, pharmacodynamics, and target engagement; however, no clinical trial information has been registered at the time of this writing.

### CSF1R (edicotinib)

Signaling via the microglial CSF1 receptor (CSF1R) is critical for microglial survival, and inhibition of CSF1R in brain has been shown to eliminate nearly all microglia in adult mice [113]. By binding to its canonical ligands (i.e., CSF1 and IL-34) on microglia, CSF1R stimulates the transition of a slow-proliferating, quiescent, anti-inflammatory phenotype to a rapidly-proliferating, activated, pro-inflammatory phenotype. Prolonged CSF1R agonism is thought to lock microglia into a maladaptive, chronic inflammatory state that can contribute to aberrant inflammation (Fig. 1). In AD mice, depletion of these pro-inflammatory microglia via CSF1R inhibitors can boost Aß clearance, downregulate cytokine expression, and preserve neuronal viability; these features have been associated with improvements in cognitive performance in some studies, but not others [114, 115]. Such benefits have been extended to non-AD models, where CSF1R inhibitors can similarly restore neuronal functioning and improve cognitive capacities in aged mice [116]. While CSF1R antagonism does appear to mitigate parenchymal amyloidosis in a 5xFAD model, increased Aß has been observed in vasculature following CSF1R inhibition, resembling CAA [114].

Edicotinib (JNJ-40346527; Johnson & Johnson), a small molecule CSF1R inhibitor that has been investigated for its potential in autoimmune diseases and cancer, has been examined in a Phase 1 trial targeting participants with MCI (a CDR = 0.5). This trial was completed in December 2021 (*n* = 54; NCT04121208) by a group at the University of Oxford. Primary outcomes included intrathecal changes in CSF1 and IL-34, and secondary outcomes included adverse events, unspecified blood and CSF biomarkers, drug levels in blood and CSF, and CSF extracellular vesicles. At the time of this writing, the status of the trial is unknown, and the results have not been published. However, given what is known about effect that CSF1R antagonism can have on vascular Aß deposits [114], and the adult-onset leukoencephalopathy caused by *CSF1R* mutations [117], caution should be advised when considering use of CSF1R antagonist in humans until a deeper understanding of CSF1R mechanisms is achieved.

### Leukotrienes (montelukast)

Montelukast (Singulair; Merck; generically available since 2012) is another small molecule being investigated for its therapeutic potential in AD. This is an FDA-approved compound that treats asthma and seasonal allergy symptoms by inhibiting LTD4-CysLT in pulmonary tissue, resulting in attenuated inflammation [118]. Inhibition of LTD4-CysLT via montelukast can mitigate Aß deposition, boost neuronal viability, enhance microglia homeostasis, prevent neuroinflammation, and preserve cognitive functioning (Fig. 1) [119–122]. A pharmacoepidemiology study (*n* = 203,473) showed that montelukast was associated with lower risk of dementia, nursing home residency, and death [123]. In a Phase 1 study (*n* = 8), IntelGenx Corp. administered montelukast via a dissolving oral strip, which increased bioavailability by 52% compared to its tablet form and enabled penetration cross the BBB, as indicated by pharmacologically active drug doses in CSF [124]. In a Phase 2 trial of clinically defined MCI and AD participants (*n* = 32; NCT03991988) led by investigators at Emory University, escalating oral doses of montelukast (10, 20, 40 mg; increased in two-week increments) were investigated for 12-months, where primary outcomes included safety parameters and secondary outcomes included cognitive performance as well as CSF biomarkers. Results suggested the drug was well tolerated, but no clear neurocognitive benefits were noted [125]. Currently, there is a Phase 2 trial (*n* = 52; NCT03402503) of montelukast led by IntelGenx Corp. across multiple sites in Canada, which recently completed enrollment. With an anticipated completion date of March 2024, the study’s primary endpoint is the global cognition composite score at 26 weeks [126]. No results have been released at the time of this writing.

### Soluble Tumor Necrosis Factor (sTNF)

As an antibody-based therapy, XPro1595 (INmune Bio) is a selective antagonist of the soluble TNF (sTNF) ligand. It heterotrimerizes with sTNF, neutralizing its bioactivity without perturbing the physiological role of membrane-bound TNF, thereby avoiding disruptions in canonical (e.g., antiviral) TNF functions (Fig. 1) [127, 128]. In rodent models of AD, aging, and other neurodegenerative diseases such as PD, the administration of XPro1595 results in brain penetrance, significant decreases in neuroinflammatory markers, improved cognitive functioning, reduced neuropathology, and enhanced neuronal functioning [128–130]. A Phase 1 trial (NCT03943264) to assess the safety of weekly XPro1595 subcutaneous injections (0.3–1.0 mg/kg, 12 weeks) among AD individuals (*n* = 20) with elevated inflammatory biomarkers was recently completed. In this study, XPro1595 treatment decreased intrathecal levels of inflammatory (CRP, YKL-40) and neurodegeneration (NfL, NRGN, pTau) biomarkers, as well as white matter free water (an MRI measure of neuroinflammation) [131]. Ongoing open label extension (OLE; *n* = 11; NCT05522387) and Phase II (*n* = 201; NCT05318976) trials will assess the efficacy of weekly XPro1595 subcutaneous injections (1.0 mg/kg, 24 weeks) among AD individuals with elevated inflammatory biomarkers, where the primary outcomes include cognitive function and plasma biomarkers (Aß, pTau181). The OLE and Phase II trials of XPro1595 have anticipated completion dates of September 2024 and May 2025, respectively.

### Other therapeutic targets

Several other immune-related compounds are discussed in the context of our Research Roadmap below and/or listed in Table 1. These compounds include complement component 1q (C1q) inhibitors such as BMS-984923 and ANX005, which can restrain aberrant microglia phagocytosis and preserve synaptic integrity despite the presence of Aß [132, 133]. Similar patterns have been observed following the pharmacological inhibition or genetic ablation of complement component 3 (C3), suggesting that the mitigation of one or more components in this innate immune signaling cascade may prove to be effective therapeutic approaches [134, 135]. Among other immune-related drug candidates, clinical trials are also investigating modulators of the Janus kinase/signal transducers and activators of transcription (JAK/STAT) pathway (sargramostim, NCT04902703; baricitinib; NCT05189106), a p38 inhibitor (MW150, NCT05194163), and PD-1 inhibitor (IBC-Ab002, NCT05551741), a broad anti-inflammatory small molecule (Lenalidomide, NCT04032626) and an antibody-based galectin inhibitor (TB006, NCT05476783).

## Research roadmap

### Preclinical therapeutics

#### CD33 inhibition

Activation of CD33 by its natural ligands, sialic acid–containing glycans, inhibits microglia from functionally adapting to dyshomeostatic conditions (e.g., Aß deposition). Thus, by blocking activation of CD33, microglia may be able to more effectively respond to AD pathology. ATLX-1088 (Alchemab Therapeutics) is in preclinical development as an antibody antagonist of the CD33 receptor. ATLX-1088 was developed following the discovery of CD33 autoantibodies among centenarians and cognitively resilient individuals [136]. Additionally, CD33 has been previously implicated in several AD genetic studies, and knockout of CD33 has been shown to augment microglia phagocytosis of Aß [137–139]. Although results have not yet been peer reviewed, data presented at a recent conference indicates ATLX-1088 may be a potent therapeutic [80]. In a multi-cell culture system (i.e., neurons, astrocytes, microglia), ATLX-1088 inhibited inflammatory cytokine production that was otherwise observed in response to LPS and interferon-γ stimulation. There are no current trials for ATLX-1088.

#### cGAS-STING therapies

Cyclic GMP-AMP synthase (cGAS) and stimulator of interferon genes (STING) together represent another innate immune pathway being examined for its therapeutic potential. cGAS-STING are key components of a DNA sensing immune response pathway that is critical for host defense. cGAS-STING stimulation leads to activation of NF-kB and the induction of type 1 interferons (IFN-I) genes, most notably cytokines IFNα and IFNß [140]. Since its discovery in 2012, cGAS-STING has been implicated in age-related inflammation and neuroinflammation, as have IFN-I genes IFNα and IFNß [141], while upregulation of the IFN-I pathway has been associated with AD [141, 142], tauopathy [143], and future cognitive decline [144], H-151 is a small molecule inhibitor of STING [145] that has been shown to suppress the induction of multiple IFN-I stimulated genes [146]. In aged mice, H-151 inhibition of STING dampens inflammatory cytokine expression, improves spatial memory, reduces microglial activation, and lowers immunoreactive hippocampal gene expression [146]. In 5xFAD mice, H-151 inhibited activation of the cGAS-STING pathway in brain tissue, dampened the expression of IFN proteins, reduced the levels of Aß_42_, Iba1 + microglial, GFAP + astrocytes, and the expression of neuroinflammatory genes, while also increasing the phagocytic activity of microglia [141, 147]. Despite compelling evidence that this pathway is implicated in AD and neurodegeneration [148–150], there are no ongoing trials for therapeutics which target the cGAS-STING axis.

#### Targeting the NLRP3 inflammasome & apoptosis speck-like protein complexes (ASCs)

Recent evidence suggests that targeting the NLRP3 inflammasome may also represent a promising therapeutic approach in AD, including results showing the deletion of its Nod-like receptor can abrogate both amyloidosis and tauopathy [33, 151]. Small molecule inhibitors such as MCC950 (Inflazome) and CY-09 (Can-Fite Biopharma) have been developed to specifically block NLRP3 inflammasome activation, effectively suppress IL-1ß production, and minimize inflammatory responses in conditions such as rheumatoid arthritis (MCC950) and multiple sclerosis (CY-09) [152, 153]. Therapies have also targeted ASCs, as studies suggest these NLRP3 inflammasome components may be released into the extracellular space by microglia following inflammasome induced pyroptosis, where they subsequently seed Aß plaques [154]. Antibodies directed against ASC specks – including AC Immune’s anti-ASC antibody, ACI-6635 – have been shown to reduce ASC-induced Aß42 aggregation in vitro, while similar administration in APP/PS1 mice can reduce plaque size and the amount of plaque-adjacent ASCs [80]. There are no current trials targeting the NLRP3 inflammasome.

### Technological innovations

**Fig. 2:**
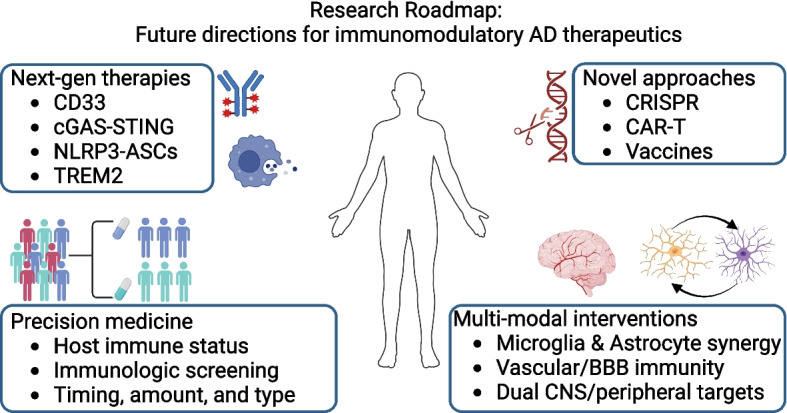
Research roadmap. Future studies to advance immunomodulatory AD therapeutics are depicted, including the continued development of next-generation therapies, the application of individualized treatment approaches, the investigation of novel therapeutic strategies, and the employment of multi-modal interventions that target a combination of immunologically relevant disease mechanisms

Novel gene therapies, cell therapies, drug delivery mechanisms, as well as next generation vaccines, may offer unique opportunities to unlock immune-related therapies for AD (Fig. 2). Perhaps the most consequential advance is CRIPSR (clustered regularly interspaced short palindromic repeats), which takes advantage of a bacterial adaptive immune system to enable low cost, accurate, and reliable modifications of nucleotide sequences [155]. Although cell-specificity and off-target effects should be considered, CRISPR strategies may offer a means of excising or modifying pathogenic microglia AD risk genes, such *CD33*, *APOE*, and *TREM2*. Another notable innovation has been the development of CAR (chimeric antigen receptor)-T cell therapy. After extraction from the blood and transduction with a customized CAR that can recognize a specific protein expressed by a distinct cancer cell type, T-cells are then infused back into the patient, where they can then use this encoded protein to locate and selectively destroy cancer cells [156]. If this technique could be adapted to antigen targeting properties of innate immune cells, bioengineered brain-honing macrophages could offer efficient degradation of specific pathological peptides like Aß. Modulation of regulatory T-cells (Tregs) may also offer opportunities to leverage immune-based cell therapies for AD. For example, Aß-specific Tregs can mitigate amyloid deposition, neuroinflammation, and cognitive deficits in APP/PS1 mice, and Treg activation with the CD3 biologic Foralumab can improve cognitive task performance in 3xTg mice despite persistent Aß levels [157, 158]. Similarly, stimulation of Treg expansion in AD patients with the interleukin-2 modulator COYA 301 can decrease intrathecal Aß levels with trending (albeit statistically non-significant) protective effects on NfL and cognitive functioning (*n* = 38; NCT06096090) [159]. Along with advances in gene and cell therapies, noninvasive focused ultrasound, which temporarily opens the BBB in precise locations, and the use of “brain shuttles”, which conjugate an existing drug molecule to high-affinity BBB ligand (e.g., a Fab fragment that binds the human transferrin receptor), could usher in a new wave of drug repurposing for compounds with potent immuno-modulatory properties that are otherwise hindered by their limited CNS penetrance [160, 161]. The renewed focus on Aß-specific vaccines may also yield unique opportunities to enhance immune-related treatments for AD. Unlike the first generation of Aß vaccines, which elicited strong T-cell responses that compromised patient health, second generation vaccines preferentially target B-cells, and are proving to be safer and more efficacious [162, 163]. Additional studies should examine if these B-cell induced benefits can be maximized by eliciting synergistic responses from other immune cell types. For example, given that non-classical monocytes along the brain endothelium express specific chemotaxis receptors (e.g., CCR2, TLR7), respond to B-cells, and differentiate into phagocytotic macrophages, vaccination with co-stimulation of CCR2 or TLR7 may lead to even greater recruitment of these protective immune cells and accelerate Aß clearance compared to vaccine treatment alone [164]. Another viable vaccine strategy could be the re-purposing of existing vaccine adjuvants that can effectively elicit protective microglia phenotypes, as is the case with Protollin, which can induce microglial activation and Aß clearance [165].

### Multi-modal therapeutics

A better understanding of how different immune mechanisms act in concert to impact disease trajectory may reveal opportunities for multi-modal immunological interventions in AD (Fig. 2). For example, because purinergic (P2X) receptor activation on microglia can halt Aß degradation, P2X antagonists may prove to be an attractive AD target; however, ATP released by activated astrocytes also functions as a potent P2X ligand [166, 167]. Thus, a theoretical drug to boost P2X-mediated degradation of Aß by microglia might be more efficacious if it was accompanied by a compound that neutralized astrocyte-derived ATP. Because the inhibition of Aß-induced glutamate dysregulation in astrocytes with levetiracetam can preserve neuronal functioning in vitro (although it’s recent Phase 2 clinical trial found null results; *n* = 8; NCT03489044), and the removal of reactive astrocytes with HT-ALZ can reduce amyloid burden and enhance cognitive performance in AD mice, future strategies should consider how potential multi-modal modulation of both astrocytes and microglia might unlock mechanisms for inhibiting AD neuropathology [168–170]*.* As evidenced by an Aß-specific-growth arrest-specific 6 (Aß-Gas6) monoclonal antibody, which can induce microglial Aβ clearance without triggering inflammatory responses or reactive gliosis in preclinical models, future strategies should also consider how the application of multiple compounds with complementary immune effects (e.g., anti-inflammation, phagocytosis) might boost resiliency to AD [171]. Along with understanding how crosstalk among specific immune cell types can enhance therapeutic efficacy in AD, a greater emphasis on dual targeting of brain and vascular mechanisms may prove to be an especially potent therapeutic strategy. Stimulating the phagocytic clearance of vascular amyloid prior to the application of Aß-targeting therapies could enhance subsequent clearance of Aß from the brain, while simultaneously mitigating ARIA risk [54]. However, therapeutic approaches aimed at reducing ARIA would likely need to do so independent of complement and perivascular macrophage activation, both of which have been shown to play a major role in ARIA development [55, 56]. Such a strategy could be particularly efficacious for *APOE*4 carriers, who are at the greatest genetic risk for AD and ARIA. Such multi-pronged strategies that leverage immune mechanisms spanning both the brain and peripheral organ systems may provide opportunities to target discrete pathogenic processes within a unified working model.

### Precision immunotherapeutics

A major challenge for the field is predicting the magnitude, direction, and type of immunomodulation that will be optimal for a given patient (Fig. 2). It is likely that a desired intervention will depend on disease stage, comorbid pathology, and underlying genetics. Many of the gene variants identified thus far suggest that genetic risk is associated with an inability of immune mechanisms to functionally adapt to neuropathogenesis, especially during the initiation phase of AD. However, later disease stages and the promotion of tauopathy by Aß may be attributed to excessive or qualitatively distinct forms of innate immune activation that might benefit from suppression. Modulation of microglia subtypes in a way that accounts for host- and disease-stage factors may therefore optimize the benefits of AD treatments. While precision medicine implies patient-specific treatment options, its application in the context of immune-related therapies for AD also requires a greater understanding of how individual variation in host immune states can influence treatment response. For example, the immune response for a given antigen can vary considerably from person to person with factors such as age, sex, immune memory, and immunosenescence accounting for further heterogeneity. Because these factors likely influence the manner in which one’s immune system responds AD pathology, it will be important to (i) understand how such between-subject variation may influence the safety and efficacy of immunomodulatory therapeutics and (ii) identify minimally invasive biomarkers that can be used to characterize relevant immunologic features and help guide therapies [172]. Immunosenescence, in particular, may represent an important source of heterogeneity for drugs that affect cell survival pathways (e.g., CSF1R and TREM2, as described above) and a therapeutic target for AD and other conditions for which neuroinflammation may drive the disease process [173, 174].

As alluded to above, biomarkers are also likely to play an important role in precision immunotherapeutics. For example, soluble forms of experimental therapeutic targets, such as sTREM2 and CD33, are detectable in blood and may be useful as theragnostic indicators for targeting TREM2 and CD33, respectively. Given that not all individuals who develop AD will have altered or abnormal immune function amenable to therapeutic intervention, immunomodulatory therapeutics should – at a minimum – be used in conjunction with biomarkers that indicate whether the targeted pathway is altered enough to benefit from intervention. Using data from completed randomized clinical trials for AD that failed to meet their primary endpoint, multiple studies have demonstrated that immunologically relevant proteins measured at study baseline can accurately predict responders and non-responders to drugs such as Celecoxib, rofecoxib, and naproxen [175, 176]. These findings highlight the potential utility of biomarker-guided patient selection for immunomodulatory therapeutics.

### Potential pitfalls

Despite their promising results and unique potential for treating AD, both acute and long-term risks of immunomodulatory therapeutics should be considered. Several repurposed compounds (e.g., the antiviral Valacyclovir) have established safety profiles, but side effects of other treatments have been documented, particularly TREM2 antibodies. As mentioned above, the development of Denali Therapeutics’ TREM2 mAb (DNL919) was halted following adverse anemic reactions, and a clinical investigation of Alector’s TREM2 mAb (AL002) was temporarily halted following cases of severe ARIA among *APOE4* homozygotes; in both instances, symptoms resolved following treatment withdrawal. Although several immunomodulatory therapeutics discussed herein have demonstrated their safety in Phase 1 trials (Table 1), comprehensive risk–benefit analyses of these treatments will require additional data from Phase 2 and 3 trails. It will be particularly important to monitor for side effects associated with other immune modulators (e.g., immunosuppressants), including acute reactions proximal to treatment administration (e.g., fever, rash, and swelling or irritation at injection sites etc.,), and long-term consequences of immunomodulation (e.g., increased prevalence of some cancers, infections etc.,) [177].

## Conclusion

Although multiple anti-Aß therapies have recently demonstrated an ability to slow the rate of AD progression, there remains an unmet need for disease modifying therapies that can safely provide further slowing and perhaps even halting of disease progression. It is now broadly accepted that immune function, both within and outside of the CNS, plays a critical role in the development of AD and therefore constitutes a viable treatment target. Some immunomodulatory therapeutics have already demonstrated positive safety profiles and evidence of potential efficacy, whether it be preclinically or in early clinical phase studies. As of 2024, for the first time, the number of immune therapeutics in the pipeline exceeds the number of those targeting amyloid [178]. The field’s optimism surrounding immunomodulatory therapies for AD is tied to multiple factors. First, many clinical and experimental stage immunomodulatory therapies are supported by highly reproducible genetic evidence. An approximate two-fold higher probability of success has been observed for drug candidates with firm genetic support (e.g., GWAS, Mendelian randomization etc.,) [179]. Second, unlike the single-target, anti-Aß therapeutics tested over the past several decades with high rates of failure, each immunomodulatory therapeutic described above is designed to engage distinct molecular targets and biological pathways via unique mechanisms of action. Biology aside, the uncorrelated and diversified nature of these individual ‘shots on goal’ may theoretically yield a higher rate of success. Lastly, because many of the promising immunomodulatory therapies are understood to modify disease progression through mechanisms that are at least partially independent of amyloid removal, there is a high likelihood that such therapies may provide an additive benefit to patients when used in combination with approved anti-Aß therapeutics.

## Data Availability

Not applicable.
